# Current Immunotherapies for Glioblastoma Multiforme

**DOI:** 10.3389/fimmu.2020.603911

**Published:** 2021-03-09

**Authors:** Boyuan Huang, Xuesong Li, Yuntao Li, Jin Zhang, Zhitao Zong, Hongbo Zhang

**Affiliations:** ^1^Department of Neurosurgery, Beijing Electric Power Hospital, Beijing, China; ^2^Department of Neurosurgery, Huizhou Third People’s Hospital, Guangzhou Medical University, Huizhou, China; ^3^Department of Neurosurgery, Zhujiang Hospital, Southern Medical University, The National Key Clinical Specialty, The Engineering Technology Research Center of Education Ministry of China, Guangdong Provincial Key Laboratory on Brain Function Repair and Regeneration, Guangzhou, China; ^4^Department of Neurosurgery, The First Affiliated Hospital, Jinan University, Guangzhou, China; ^5^Department of Neurosurgery, Jiujiang Hospital of Traditional Chinese Medicine, Jiujiang, China

**Keywords:** immunotherapy, glioblastoma multiforme, glioma, vaccines, checkpoint inhibitors

## Abstract

Glioblastoma multiforme (GBM) is the most common and aggressive malignant tumor found in the central nervous system. Currently, standard treatments in the clinic include maximal safe surgical resection, radiation, and chemotherapy and are mostly limited by low therapeutic efficiency correlated with poor prognosis. Immunotherapy, which predominantly focuses on peptide vaccines, dendritic cell vaccines, chimeric antigen receptor T cells, checkpoint inhibitor therapy, and oncolytic virotherapy, have achieved some promising results in both preclinical and clinical trials. The future of immune therapy for GBM requires an integrated effort with rational combinations of vaccine therapy, cell therapy, and radio- and chemotherapy as well as molecule therapy targeting the tumor microenvironment.

## Introduction

Glioblastoma multiforme (GBM) is the most common and aggressive primary malignant tumor in the central nervous system (CNS) in adults (1). It is mainly classified into two groups: isocitrate dehydrogenase (IDH)-wildtype GBM, which has been previously referred to as primary GBM and represents about 90% of cases, and IDH-mutant GBM, which is developed from a lower-grade diffuse glioma and represents about 10% of cases. The current standard treatments for GBM include a combination of surgical resection, radiation, and chemotherapy. At present, there are only two drugs approved by the FDA to treat GBM *via* systematical administration: temozolomide (TMZ) for the treatment of newly diagnosed GBM (ndGBM) and bevacizumab for the treatment of recurrent GBM (rGBM) (2, 3). Unfortunately, current therapeutic approaches have very limited impact on improving the prognosis of GBM patients, showing 15 months of median survival and less than 5% of a 5-year survival rate (1). Thus, opportunities and challenges remain in finding more efficient treatments against GBM.

Immunotherapy, which manipulates the immune system to attack tumor cells with minimal adverse effects and prevents tumor remission, has drawn extensive attention (4). However, there are still challenges that need to be overcome in the development of immunotherapy for GBM. The CNS is considered to be an “immune-privileged” organ, attributed to the lack of lymphatic involvement and the selectivity of the blood–brain barrier (BBB) to immune cells (5). Antigens in the brain can still drain into the cervical lymph nodes through lymphatic vessels in the dura and meninges (6). Moreover, microglia, as the brain’s resident immune cells, can function as potential antigen presentation cells (APCs), and T cells are activated in the cervical lymph nodes entering the brain parenchyma through the cerebrospinal fluid (CSF) (7). These observations suggest that the brain is immune privileged to a certain degree, and blood-derived immune cells are not completely precluded from the brain (8, 9). Moreover, GBM cells can exert local immunosuppressive effects in many ways. On the one hand, GBM cells themselves can secrete various protumor cytokines and/or chemokines, which can influence macrophage polarization, promote regulatory T cell (Treg) recruitment, and inhibit dendritic cell (DC) maturation and natural killer (NK) cell function. On the other hand, GBM cells can express immunosuppressive molecules, such as programmed cell death protein 1 ligand (PD-L1), which can prevent T cell proliferation and activation (10). In spite of these challenges, immunotherapy for GBM still obtains considerable achievements, which have given rise to a number of clinical trial investigations. Increasing immunotherapeutic approaches for GBM treatments have also been established. In this review, we present an overview of the current immunotherapy for GBM, including peptide vaccines, DC vaccines, chimeric T-cell receptors, checkpoint inhibitors, and oncolytic virotherapy.

## Peptide Vaccines

Peptide vaccines are about 8–30 amino acids in length. They are designed to encompass tumor-specific antigens (TSA), which derive from mutations only expressed in tumor cells but are absent in normal cells, or tumor-associated antigens (TAA), which derive from overexpressed normal proteins that are present in both tumoral and normal tissue. Unlike other solid tumors, GBM is notorious for possessing a relatively low level of mutation, resulting in only a minority of mutations used as TSA (11). At present, the peptide vaccines under investigation in GBM include rindopepimut (12), IMA950, and isocitrate dehydrogenase 1 (IDH1). The epidermal growth factor receptor variant III (EGFRvIII), with a mutated deletion in 20%–30% of tumors, is the most relevant and uncontroversial TSA for GBM. Thus, targeting EGFRvIII as a primary example of TSA-based peptide vaccines has been extensively investigated in the immunotherapy against GBM. In a phase II clinical trial, 65 patients with EGFRvIII-positive GBM were administrated with rindopepimut as well as with standard adjuvant TMZ (13) (**Table 1**). As a result, a progression-free survival (PFS) at 5.5 months of 66% and a median overall survival (OS) of 21.8 months were observed (13). In another phase II clinical trial, bevacizumab plus rindopepimut or a placebo were tested in rGBM patients, indicating that PFS at 6 months was 27%, and the median OS was 12 months, which is significantly improved compared with the control group of a PSF at 6 months of 11% and a median OS of 8.8 months (14). Following these achievements, a large, randomized, double-blind, placebo-controlled phase III clinical trial, enrolling 745 patients with ndGBM was terminated early after showing no significant improvement in the median OS. However, the data demonstrate patients with decent humoral immune responses (15) (**Table 1**). Notably, lost expression of EGFRvIII (antigen escape) was observed in the control arm to a similar degree as that of the treatment arm, which challenges the notion that therapies targeting EGFRvIII should be responsible for the outgrowth of EGFRvIII-deficient GBM cells (16, 17). In addition, this study also highlights that targeting a single tumor antigen may not be sufficient enough to induce durable antitumor responses.

**Table 1:** Completed representative clinical trials of immunotherapy.

Immunotherapy approach	Phase	Sample size	PFS(m)	OS(m)	Characteristics
**Vaccine**					
Rindopepimut (15)	III	745	8	20.1	First clinical trial of an EGFRvIII-targeted therapy for newly diagnosed GBM
IMA950 (18)	I	45	NR	15.3	Evaluated the most biologically effective and clinically feasible
DCs vaccine (110)	II	26	12.7	23.4	Vaccine schedule design to deliver vaccine before radiation therapy
CMV pp65 DCs vaccine (32)	I	11	25.3	41.1	Provides evidence for targeting the association between CMV and GBM
SurVaxM peptide vaccine (111)	I	9	17.6	86.6	First study of SurVaxM in recurrent malignant gliomas
CDX-110 (13)	II	65	5.5	21.8	Multi-center phase II trials of CDX-110 with TMZ and radiation in GBM
HSPPC- 96 vaccine (112)	I/II	41	4.5	9.5	Establishes HSPPC-96 vaccine for recurrent malignant gliomas
GSCs derived mRNA transfected DCs vaccine (113)	I	20	23.1	25.5	First study targeting GSCs demonstrating feasibility, safety of an active immunotherapy targeting GSCs
**Adaptive T cells**					
IL13Rα2-CAR-T cells (57)	I	3	NR	11	First-in-human pilot safety and feasibility trial evaluating CAR-T cell targeting IL13Rα2 for recurrent GBM
INNOCELL Immuncell-LC (114)	III	180	8.1	22.5	First prospective, multicenter, randomized,controlled study of cytokine-induced killer cells therapy for newly diagnosed GBM
CMV-specific T cells (115)	I	19	8.2	13.3	First clinical trial of adoptive CMV-specific T cells for recurrent GBM
HER2-CAR-CMV-T cells (61)	I	16	3.5	24.5	First phase I trial of autologous HER2-CAR-CMV-T cells in GBM
**Checkpoint Inhibitor**					
Pembrolizumab (83)	II	80	4.1	8.8	First trial of pembrolizumab with Bevacizumab in recurrent GBM
Ipilimumab (116)	II	72	NR	7vs 4	First open label study of ipilimumab in melanoma patients with brain metastases
Nivolumab	III	369	1.5	9.8	First large randomized clinical trial of PD-1 inhibition in GBM

GBM, glioblastoma; CAR-T, chimeric antigen receptor T cells; OS, overall survival; PFS, progression-free survival; HSPPC-96, heat shock protein peptide complexes 96; CMV, cytomegalovirus; EGFR vIII. epidermal growth factor receptor variant III; HER-2, human epidermal-growth-factor receptor 2; GSCs, glioma stem cells; CMV pp65, cytomegalovirus phosphoprotein 65 RNA; DCs dendritic cells.

IMA950 is a novel therapeutic vaccine that includes nine synthetic tumor-associated HLA-A2-restricted peptides (TUMAP), two MHC class II–binding peptides, and one HLA-A2-restricted HBV-derived peptide, and the last one was also used as a marker of vaccine immunogenicity. IMA950 can trigger the stimulation of TUMAP-specific cytotoxic T cells, leading to the destruction of malignant tumor cells. In a phase I trial, patients diagnosed with ndGBM after tumor resection were injected intradermally with IMA950 either prior to or just after the initiation of chemoradiotherapy. The majority of patients were found to respond well with a PFS at 6 months of 74% and a median OS of 15.3 months (18) (**Table 1**). In a recently completed phase I/II trial, IMA950 with vaccine adjuvant poly-ICLC in combination with TMZ were tested in 19 patients (16 with GBM and three with anaplastic astrocytoma). Patients from the overall cohort showed a median OS of 21 months from the date of surgery, compared with the GBM-only cohort of 19 months. PFS of patients from the overall cohort were 93% and 56% at 6 and 9 months, respectively (19). As for rGBM, however, IMA950 has no benefit in any preclinical trial. In a previous clinic trial, patients with recurrent high-grade gliomas who were administrated bevacizumab with the IMA950/poly-ICLC peptide vaccine did not show improved OS and PFS compared to nonvaccinated patients (20).

IDH1 mutations can be found in nearly 90% of low-grade gliomas, and more than 90% of IDH1 mutations contain an arginine-to-histidine switch at position 132 (IDH1^R132H^). In GBM, IDH1 mutations can predict whether the tumors are secondarily developed from lower-grade gliomas because IDH1 mutations are rarely found in primary GBM. This high-frequency neoantigen is expressed in more than 70% of rGBMs, which can induce the formation of the oncometabolite 2-hydroxyglutarate and the inhibition of NADPH production (21, 22). Preclinical studies suggest peptide vaccines spanning the IDH1 mutation, may elicit IDH1^R132H^-reactive CD4^+^ and CD8^+^ responses for antitumor (23). A phase I clinical trial at Duke University is ongoing in which the intradermal IDH1 peptide vaccine is tested in IDH1-positive grade II primary brain tumors (ClinicalTrials.gov identifier: NCT02193347). In another phase I trial, the safety of the IDH1 peptide vaccine for high-grade gliomas was also being evaluated. This study was completed in 2019 (ClinicalTrials.gov identifier: NCT02454634). Data collection is ongoing, and the therapeutic efficiency of IDH1 vaccines will be further estimated.

To date, several peptide vaccine strategies are shown to have safe and efficient profiles in phase I and II clinical trials, and some vaccines have significantly improved patient survival compared with historical controls. However, supportive data from phase III trials are still lacking. Although a phase III clinical trial on the EGFRvIII-based vaccine has failed in ndGBM patients, this vaccine could still induce decent humoral immune responses (15). Accordingly, more phase III trials on the peptide vaccine are required to support the therapeutic potential of peptide vaccines in GBM treatment. In addition, the single-antigen targeted strategy may lead to antigen escape due to high heterogeneity in the tumor. Therefore, alternative vaccine approaches are needed to target multiple tumor neoantigens. Heat shock protein (HSP) peptide complexes 96 (HSPPC-96) is one solution to handling this problem. HSPPC-96 is a primary resident chaperone of the endoplasmic reticulum, which can be internalized into APCs for efficient class I and II MHC-mediated presentation of tumor peptides (24). In a phase I clinical trial, an HSPPC-96 vaccination induced a tumor-specific peripheral immune response in 11 of 12 high-grade glioma patients (25). A subsequent open-label phase II multicenter clinical trial in surgically resectable rGBM patients treated with HSPPC-96-loaded antigens, which were extracted from patient-derived glioma tissue, showed an impressive median OS of 42.6 weeks and a 6-month OS of 29.3%, respectively (26). These results have sparked multiple ongoing clinical trials: NCT00905060, a completed phase II trial exploring the application of autologous HSPPC-96 following tumor resection and adjuvant RT and TMZ in ndGBM, and NCT01814813, a multi-institutional trial investigating the safety, tolerability, and efficacy of HSPPC-96 combined with bevacizumab in rGBM patients.

## DC Vaccines

DCs are able to present tumor antigens to CD4^+^ and CD8^+^ T cells to stimulate an immune response. Therefore, vaccines based on DCs represent another immunotherapeutic approach. This type of vaccine is typically produced through the *ex vivo* generation of DCs harvested from patients. The isolated DCs are stimulated by either tumor antigens or mRNA-expressing MHC molecules before administration (27, 28). Currently, there are strategies for DC vaccines exposed to either single specific antigens or multiple tumor antigens. In a phase I trial, seven patients with high-grade gliomas were administered Wilms’ tumor 1 (WT1)-pulsed autologous DCs. Five patients showed stable clinical responses, and the OS was 12.3 months in the cohort after the first DC vaccination (29). Cytomegalovirus phosphoprotein 65 RNA (CMV pp65) is also incorporated into DC vaccines because CMV nucleic acids and proteins are found in both primary and recurrent GBM (30). In another phase I trial, patients with ndGBM were administered pp65-specific DCs in combination with preconditioning using tetanus-diphtheria toxoid (Td). It achieved a promising PFS of 15.4–47.3 months and OS of 20.6–47.3 months (31). Batich et al. applied dose-intensified TMZ followed by a CMV pp65 DC vaccine to treat 11 ndGBM patients in a phase I trial. Both median PFS and OS are longer than predicted ones (32) (**Table 1**). Currently, a randomized phase II trial involving a CMV pp65 DC vaccine is recruiting ndGBM patients (ClinicalTrials.gov identifier: NCT02465268) (**Table 2**). Another similar clinical trial on IDH1 DC vaccine for glioma treatments is also under investigation in China (ClinicalTrials.gov identifier: NCT02771301).

**Table 2:** Ongoing clinical trials involving DC vaccine, checkpoint inhibitor and CAR-T.

NCT number	Phase	Name of trial	Status	Patient enrolled
**DC Vaccine**				
NCT02649582	I/II	Adjuvant DC-immunotherapy Plus TMZ in GBM Patients	Recruiting	20
NCT02709616	I/II	Personalized Cellular Vaccine for Glioblastoma	Recruiting	20
NCT01567202	II	Study of DC Vaccination Against Glioblastoma	Recruiting	100
NCT02772094	II	Dendritic Cell-Based Tumor Vaccine Adjuvant Immunotherapy Human GBM	Ongoing	50
NCT02366728	II	DC Migration Study for Newly-Diagnosed GBM	Recruiting	100
NCT02465268	II	Vaccine Therapy for the Treatment of Newly Diagnosed Glioblastoma Multiforme	Recruiting	150
NCT01204684	II	Dendritic Cell Vaccine for Patients With Brain Tumors	Ongoing	60
NCT02754362	II	A Toll-like Receptor Agonist as an Adjuvant to TAA Mixed With Montanide ISA-51 VG With Bevacizumab for Patients With Recurrent GBM	Recruiting	30
NCT03395587	II	Efficiency of Vaccination With Lysate-loaded Dendritic Cells in Patients With Newly Diagnosed Glioblastoma	Recruiting	136
NCT03400917	II	Autologous Dendritic Cells Loaded With Autologous TAA for Treatment of Newly Diagnosed GBM	Recruiting	55
**Checkpoint Inhibitor**				
NCT02530502	I/II	Radiation Therapy With TMZ and Pembrolizumab in Treating Patients With Newly Diagnosed GBM	Ongoing	4
NCT02337686	II	Pharmacodynamic Study of Pembrolizumab in Patients With Recurrent GBM	Ongoing	18
NCT02337491	II	Pembrolizumab +/- Bevacizumab for Recurrent GBM	Ongoing	80
**CAR-T**				
NCT01454596	I/II	CAR-T Cell Receptor Immunotherapy Targeting EGFRvIII for Patients With Malignant Gliomas Expressing EGFRvIII	Recruiting	107
NCT02617134	I/II	CAR-T Cell Immunotherapy in MUC1 Positive Solid Tumor	Recruiting	20
NCT02839954	I/II	CAR-pNK Cell Immunotherapy in MUC1 Positive Relapsed or Refractory Solid Tumor	Recruiting	10
NCT02208362	I	Genetically Modified T-cells in Treating Patients With Recurrent or Refractory Malignant Glioma	Recruiting	135
NCT02713984	I/II	A Clinical Research of CAR T Cells Targeting HER2 Positive Cancer	Recruiting	60
NCT02209376	I	Autologous T Cells Redirected to EGFRVIII-With a CAR in Patients With EGFRVIII^+^ Glioblastoma	Ongoing	12
NCT02664363	I	EGFRvIII CAR T Cells for Newly Diagnosed GBM	Recruiting	48
NCT02844062	I	Pilot Study of Autologous Anti-EGFRvIII CAR T Cells in Recurrent Glioblastoma Multiforme	Recruiting	20
NCT01109095	I	CMV-specific Cytotoxic T Lymphocytes Expressing CAR Targeting HER2 in Patients With GBM	Ongoing	16
NCT02442297	I	T Cells Expressing HER2-specific CAR for Patients With Glioblastoma	Recruiting	14
NCT02937844	I	Pilot Study of Autologous Chimeric Switch Receptor Modified T Cells in Recurrent GBM	Recruiting	20

GBM, glioblastoma; CAR-T, chimeric antigen receptor T cells; TMZ, temozolomide; HSPPC-96, heat shock protein peptide complexes 96; CMV, cytomegalovirus; TAA, tumor-associated antigen; EGFR vIII, epidermal growth factor receptor variant III; HER-2, human epidermal-growth-factor receptor 2; CMV pp65, cytomegalovirus phosphoprotein 65 RNA.

In addition, there are also DC vaccines exposed to multiple tumor antigens to induce a more robust immune response. In a phase I clinical study, an autologous DC vaccine pulsed with class I peptides from TAA highly expressed on gliomas and a cancer stem cell population (ICT-107) were administered to 15 ndGBM patients. Median PFS was 16.9 months and median OS was 38.4 months. It is also worth noting that six patients showed no evidence of tumor recurrence in a follow-up of 40.1 months (33). In another phase I/II trial, patients with recurrent glioma were administered α-type 1 polarized DCs loaded with EphA2, IL13Rα2, YKL-40, and gp100 and combined with poly-ICLC. It was observed that nine of 22 patients achieved PFS lasting at least 12 months, and one rGBM patient exhibited a sustained complete response (34). Recently, a novel DC vaccine, called DCVax-L, has been prepared from tumor lysate. In a phase I/II clinical trial, a DC vaccine was prepared with the patient’s own tumor cells prior to administration to the patients. Sixteen GBM patients were enrolled in this trial. The data show that median and 5-year survival were 525 days and 18.8%, respectively (35). A randomized phase II trial on DCVax-L and nivolumab in rGBM patients is ongoing (ClinicalTrials.gov identifier: NCT03014804). Another randomized phase III trial on DCVax-L is currently underway in 348 GBM patients (ClinicalTrials.gov identifier: NCT00045968). Given that DC cocultured with tumor lysate for the generation of DCVax-L, this kind of vaccine should be more efficient in the elimination of tumor cells because it is able to target more tumor-related antigens. However, theoretically there is also a high risk that it may cause an autoimmune response. Therefore, it still remains a challenge for researchers to choose a suitable tumor lysate for the generation of DC vaccines regarding the high heterogeneity of GBM. There is still much work that needs to be done to understand the influences of tumor genotypes and microenvironments on DC vaccine production to prevent the undesired autoimmune response during administration.

## Adoptive T Cell Therapy

The functional advantage of adoptive T cell therapy lies in its ability to harvest, train, and expand autologous T cells which are then transfered back into patients (36). The primary forms of adoptive T cell therapy can be generally classified as tumor-infiltrating lymphocytes (TILs), T-cell receptor (TCR) treatment, and chimeric antigen receptor T (CAR-T) cells. The application of TILs requires highly accessible and immunogenic tumor cells; however, only melanoma can meet sufficient expansion of TILs from their respective tumor samples (37). In a prospective pilot study including six rGBM, locally infused autologous TILs did not show powerful cytotoxicity against the autologous tumor (38). Apart from the desire for improvement in expansion of brain tumor–derived TILs, this study also implied the significance of maintaining autologous TIL activation within the brain TME. TCR treatment was the first successful application of adoptive T cell therapy that utilized autologous T cells transduced with human TCR recognizing a melanoma antigen recognized by T cells 1 (MART-1) to treat patients with metastatic melanoma (39). As far as gliomas are concerned, however, no clinical trials based on TCR-T cell therapies have been initiated. The little progress made in TILs and TCR against gliomas force researchers to seek other ways, and the efforts to overcome MHC restriction result in the development of CAR-T cell therapy.

**Figure 1:**
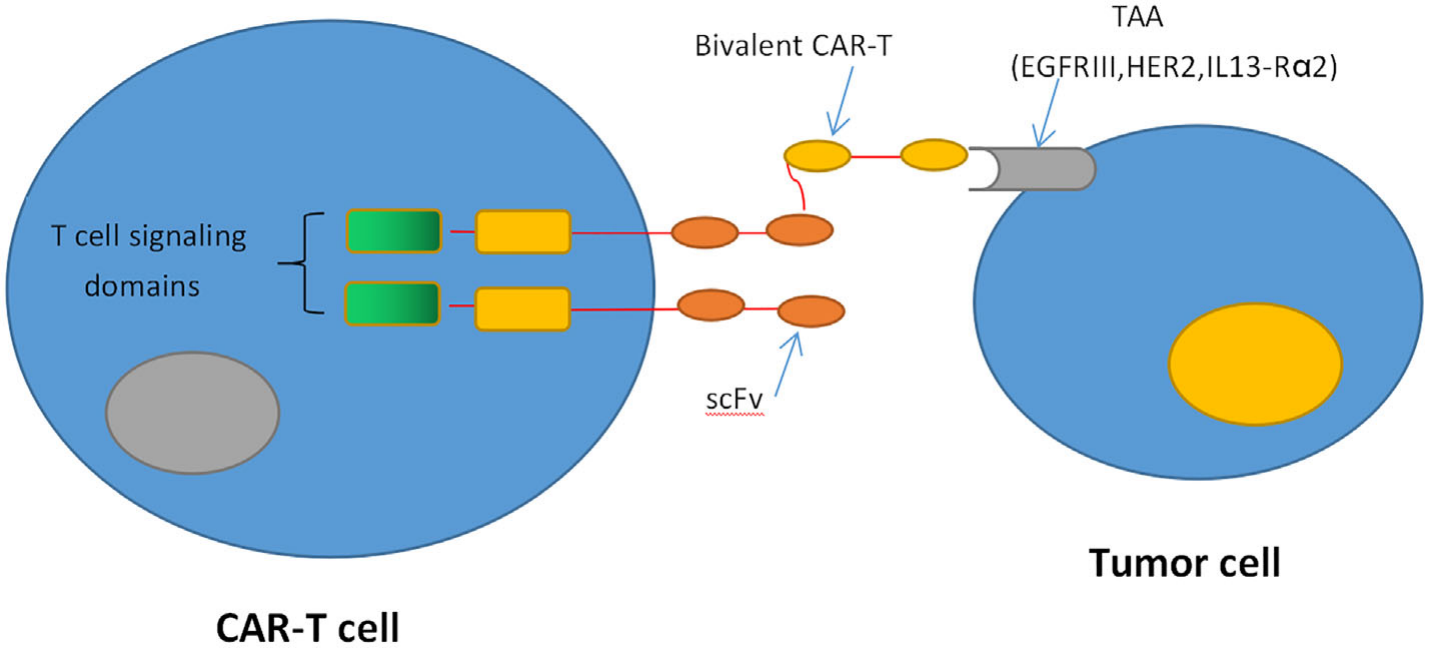
CAR T-cell therapy. CAR-T cells produce an artificial T cell receptor that has big tumor-specific surface antigens.

Recently, genetically engineered T cells expressing chimeric antigen receptors (CARs) to recognize specific tumor antigens have brought in a new era of cancer immunotherapy. CARs are artificial fusion proteins that incorporate an intracellular T-cell signaling domain that consists of one or more single-chain variable fragments (scFv) and an extracellular antigen-recognition domain to target specific neoplastic cells. These complex domains include CD28, CD3ζ, 4-1BB, or OX40 derived from the same part of CD28/CD8 or a corresponding domain of T-cell receptors (TCRs) (40, 41) (**Figure 1**). In addition to being endowed with a specific affinity to TSAs or targets of interest, CAR-T cells can be stimulated without MHC involvement and prevent the challenges associated with adoptive T-cell transfer (42, 43). Currently, CD19-specific CAR-T cells have induced sustained and durable antitumor immune responses in patients with multiple myeloma, acute and chronic lymphocytic leukemia, and refractory diffuse large B-cell lymphoma (DLBCL) (44–49). These encouraging results have prompted FDA approvals of two therapies: CTL019, a treatment for patients younger than 25 with relapsed or refractory B-cell precursor acute lymphoblastic leukemia, and another CD19-targeted CAR T-cell treatment, axicabtagene ciloleucel, for patients with failed DLBCL for at least two prior therapies (50, 51). Inspired by the success in blood tumors, increasing interest has focused on the treatments of CAR-T cells against GBM. These CAR-T cells mainly target the following antigens: EGFRvIII, IL-13Rα2, and HER2. EGFRvIII is abundantly expressed in approximately 30% of GBM to enhance glioma cell proliferation, angiogenesis, and invasiveness (52). In preclinical studies, CAR-T cells targeting EGFRvIII could effectively infiltrate to tumor sites and suppress the growth of glioma xenografts in murine models (53). In a human clinical trial, EGFRvIII-targeting CAR-T cells showed feasibility and safety in the treatment for 10 rGBM patients without toxicity or cytokine release syndromes (54). It demonstrates that transient expansion of EGFRvIII-targeting CAR-T cells could be detected in peripheral blood of all patients. The median OS was approximately 8 months, and one patient experienced residual stable disease at 18 months (54). The promising clinical trials are still ongoing to assess the efficiency of this approach (**Figure 1**).

Another target of CAR-T cell treatment for GBM patients is IL-13Rα2, which presents in more than 75% of GBM tumors associated with tumor invasiveness and poor prognosis (55, 56). As the first CAR-T targeting IL-13Rα2 therapy, the feasibility and safety of IL13-zetakine CD8^+^ CTL against rGBM have been evaluated by Brown et al. In this trial, intracranial delivery of the IL13-zetakine^+^ cytotoxic T lymphocytes (CTL) into the resection cavity was well tolerated in three patients. A transient antiglioma response was observed in two patients (57) (**Table 1**). In a following report, CAR T-cells targeting IL-13Rα2 incorporated with costimulatory immunoreceptor CD137 were initially delivered into the resection cavity of grade 3 or higher GBM. Consequently, regression of all intracranial and spinal tumors was observed without any toxic effects. Moreover, a robust increase of inflammatory cytokines and chemokines in the CSF with limited CAR T-cell accumulation and expansion was also found. Eventually, this clinical response lasted for 7.5 months after the initiation of CAR T-cell therapy (58).

Human epidermal growth factor receptor 2 (HER2) is a transmembrane tyrosine kinase receptor overexpressed in 80% of GBM. It is identified as an independent unfavorable prognostic indicator for GBM patients (59, 60). Considering that HER2 is also expressed in normal tissues, there is a theoretical high risk of off-target toxicity resulting from HER2-targeting CAR-T cells. Intriguingly, a phase I clinical trial demonstrated the feasibility and safety of HER2-targeting CAR-T cells, which were well-tolerated in 17 patients with progressive HER2-positive GBM without any dose-limiting toxic effects (61) (**Table 1**). The median OS was 11.1 months (95% CI, 4.1-27.2 months) from the first T-cell infusion and 24.5 months (95% CI, 17.2-34.6 months) from diagnosis. Three patients had no progression between 24 to 29 months (61).

Although the results from these studies are encouraging, CAR-T cells targeting a single antigen may still inevitably lead to antigen escape. To deal with this intractable dilemma, CAR-T cells targeting multiple tumor antigens have been established to overcome the heterogeneity of GBM. Hegde et al. created CAR-T cells expressing a HER2-binding scFv and an IL-13Rα2-binding IL-13 mutein, which could efficiently recognize and kill either HER2 or IL-13Rα2 positive tumor cells (62). These bispecific CAR-T cells are more sustainable and capable of improving the survival in GBM murine models and mitigating antigen escape (62). Taking this approach one step further, the same research group generated trivalent CAR-T cells targeting HER2, IL-13Rα2, and EphA2, which could overcome the interpatient variability and capture nearly 100% of tumor cells. In a murine model, the trivalent CAR-T cells exhibited superior antitumor efficacy. It significantly inhibited tumor growth and improved animal survival compared with biCAR-T cells or single CAR T-cells (63).

CAR T-cell therapy in GBM has just begun. Preliminary results demonstrate its feasibility and safety, and bi- or tri-CAR-T cells may be a promising strategy for the intractable dilemma of antigen loss. However, several problems and challenges in solving CAR-T treatment still exist.. First, T-cell proliferation and persistence is still a limitation for solid tumor treatment because the peripheral blood is not the therapeutic site. It also raises a related issue regarding whether precondition of lymphodepleting, which has been approved as a standard in CAR-T treatment of hematologic malignancies, is able to improve CAR T-cell expansion and efficacy in GBM (64, 65). Although it has not been reported to use lymphodepleting preconditioning in ndGBM (54, 57, 58), rGBM patients often accept “lymphodepletion” before CAR-T treatment due to the effects of standard radiation and TMZ (66). Another issue that needs to be addressed is the immunosuppressive TME. The TME of GBM can present many obstacles to CAR-T cells, including immunosuppressive immune cells, tumor-derived soluble factors and cytokines, and physical and metabolic barriers (67, 68). Therefore, intensive investigations are urgently needed to improve the efficacy of CAR-T treatment in GBM patients.

## Checkpoint Inhibitors

Immune checkpoints are the coinhibitory molecules that could attenuate the intensity and duration of T-cell-mediated immune responses to maintain self-tolerance and prevent uncontrolled inflammatory responses. Currently, the most well-studied coinhibitory molecules in hematologic and solid tumors include cytotoxic T-lymphocyte antigen 4 (CTLA-4), programmed cell death protein 1 (PD-1) and its ligand PD-L1, T-cell immunoglobulin and mucin domain 3 (TIM-3), and indoleamine 2,3-dioxygenase-1 (IDO1).

**Figure 2:**
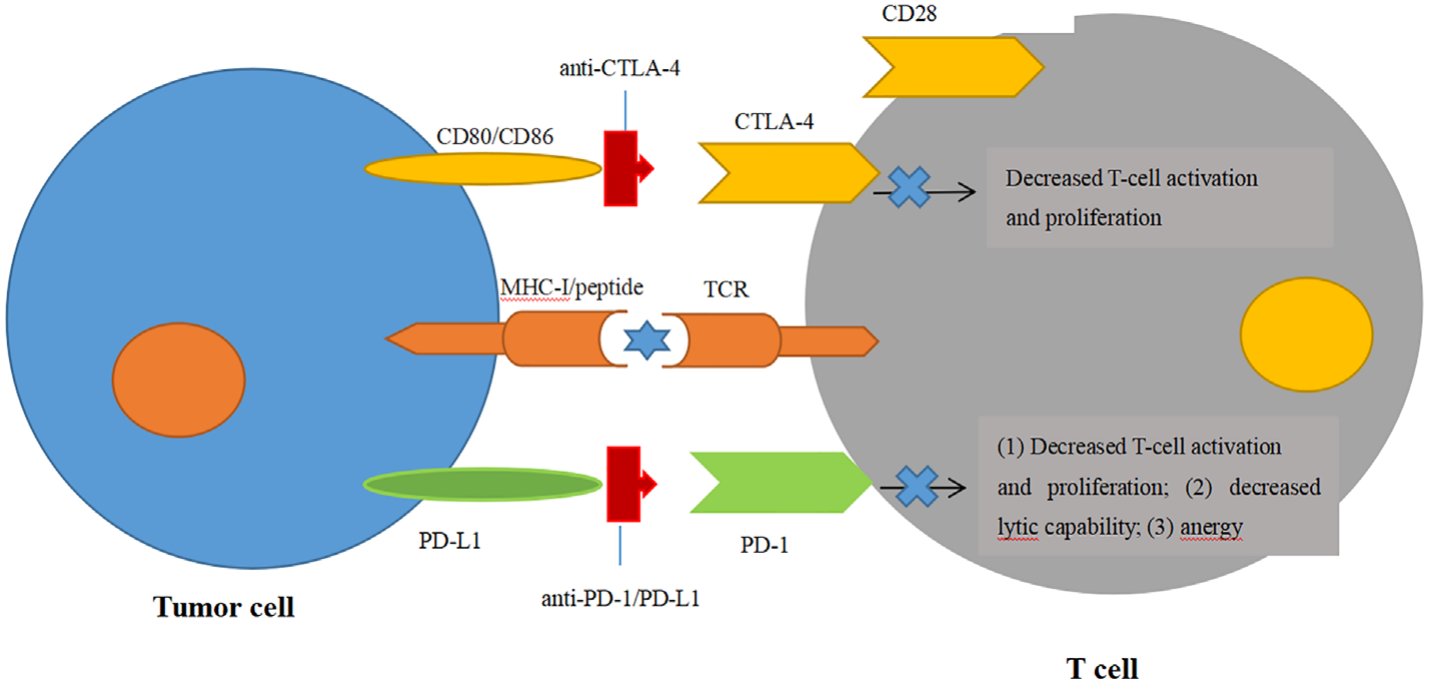
Blockade of immune checkpoint inhibitors. Engagement of CTLA-4 with its ligands CD80/CD86 can prevent the ligands binding to the T-cell activation and proliferation. Engagement of PD-1 with one of its ligands, PD-L1, can decrease the T-cell tumor lytic capacity and induces T-cell anergy.

CTLA-4 is one of the most extensively studied immune checkpoint inhibitors, and it suppresses T-cell stimulation by competing with the costimulatory molecule CD28 for binding its ligands CD80 and CD86 (69, 70) (**Figure 2**). Ipilimumab (trade name Yervoy) was the first FDA-approved checkpoint for immunotherapy targeting CTLA-4 applied in metastatic melanoma and now approved for several solid tumors. In murine glioma models, blockade of CTLA-4 could induce tumor regression and promote long-term survival without eliciting experimental allergic encephalomyelitis (71). For GBM, combinatorial blockade of CTLA-4 and PD-1 were demonstrated to cure 75% of immunocompetent murine GBM models even against advanced, later-stage tumors (72). Until now, blockade of CTLA-4 could lead to robust antitumor immunity only at the preclinical stage. Although there has been no published data on CTLA-4 inhibitors solely treating GBM yet, some clinical trials are currently ongoing to evaluate CTLA-4 inhibitors in GBM combined with other therapeutic agents, such as VEGF inhibitors, checkpoint inhibitors, tumor treating fields, and radiation therapy (73).

PD-1, an immunoglobulin receptor belonging to the extended CD28/CTLA-4 family of T-cell regulators, is expressed on activated T, B, myeloid, and NK cells. It binds to the ligands PD-L1 and PD-L2 (74). The PD-1/PD-L1 axis is proven to be the major negative regulation of CTL in the TME, whose protumor function, including suppression of T-cell activation and infiltration, is inhibiting the secretion of pro-inflammatory factors and inactivation of TCR signaling (74, 75) (**Figure 2**). Owing to the success of antibodies targeting the PD-1/PD-L1 axis in the clinical trials against advanced melanoma, monoclonal PD-1 antibodies (Pembrolizumab and Nivolumb) were approved by the FDA for the treatment of melanoma, non-small cell lung cancer (NSLC), and other solid tumors (76–80). For GBM, the therapeutic effects of PD-1/PD-L1 antibodies remain largely elusive. In a preclinical study, the combination of PD-1 antibody and radiotherapy achieved a twofold increase in median survival in GL261 glioma mouse models, and 15%–40% of mice gained long-term survival compared with a single treatment (81). In another preclinical trial, the combination of a DC vaccine and PD-1 antibody achieved long-term survival in intracranial glioma tumor-bearing mice that were solely dependent on CD8+ T cells (82). Moreover, this combination of a DC vaccine and PD-1 antibody also resulted in the upregulation of homing integrin and immunologic memory markers on TILs (82). These encouraging preclinical studies prompted the first large phase III clinical trial of PD-1 checkpoint blockade in rGBM through the comparison of nivolumab monotherapy with standard care using bevacizumab (NCT02017717). Although the median OS was comparable between nivolumab and bevacizumab among the overall enrolled patients, this trial was still closed in 2017 on account of failing to meet the primary OS endpoint (83) (**Table 1**). Another phase III randomized trial, CheckMate 548, was processed to evaluate the effects of nivolumab with or without radiation therapy and TMZ in O6-methylguanine-DNA methyltransferase (MGMT)-methylated ndGBM patients. This study has also failed to achieve the endpoint for the inability of nivolumab concomitant with radiation therapy and TMZ to improve the median OS (84). Another similar phase III trial, CheckMate 498, for patients with MGMT-unmethylated tumors also declared that nivolumab combined with TMZ failed to improve patients’ median OS. Although nivolumab has not yet shown efficiency in clinical trials, other antibody therapies targeting the PD-1/PD-L1 axis have emerged in clinical trials. Pembrolizumab, another PD-1 antibody, was tested as neoadjuvant or adjuvant-only therapy in 35 surgically resectable rGBM patients in a single-arm phase II clinical trial. Patients accepting pembrolizumab showed a statistically significant increase in OS with a median value of 417 days compared with those in the adjuvant group with 228.5 days. PFS in the neoadjuvant group was also significantly increased over the adjuvant group (99.5 days vs. 72.5 days). The study also found that neoadjuvant anti-PD-1 blockade was related to an upregulation of the IFN-γ responsive gene signature and a declined cell cycle–related gene signature in the tumor (85). In a single-arm phase I trial, pembrolizumab accompanied by hypofractionated stereotactic irradiation and bevacizumab were well tolerated in 23 rGBM patients. More than half of the patients achieved durable objective responses, and 64% of the patients were still alive within 12 months (86). Another phase I trial on combinatorial pembrolizumab with bevacizumab (NCT02337491) in rGBM patients showed a median OS of 8.8 months and PFS of 4.1 months (https://clinicaltrials.gov/ct2/show/results/NCT02337491) (**Table 2**). Additionally, durvalumab (MEDI4736), a humanized PD-Ll monoclonal antibody, is currently being tested in a multicenter phase II trial combined with radiotherapy and bevacizumab in GBM patients (NCT02336165) (87). It is striking that one patient obtained a long-period OS of 86 weeks (87). In contrast to the monotherapy by PD-1/PD-L1 inhibitors with few successes, combinatorial therapy of PD-1/PD-L1 antibodies with radiation therapy and/or chemotherapy seem more promising in the clinical trials against GBM.

In addition to CTLA-4 and PD-1/PD-L1 therapy, another two checkpoint targets have received researcher interest. TIM-3, an immunosuppressive receptor expressed on T cells, Tregs, DCs, NK cells, and macrophages, can promote T-cell exhaustion similar to PD-1 (88, 89). There are ongoing clinical trials testing TIM-3-targeted antibodies in solid tumors (NCT02608268, NCT02817633) and hematological malignancies (NCT03066648). IDO1 is a cytoplasmic enzyme promoting tryptophan catabolism through the kynurenine pathway. It is demonstrated that depletion of IDO1 can suppress T-cell function and elevate expression of IDO1 in a tumor, which is correlated with poor prognosis in GBM patients (90, 91). So far, there are some clinical trials evaluating IDO1 inhibitors in melanoma (92) and breast cancer (NCT01792050) but none showing a survival benefit. There is a phase I clinical trial including various treatments, such as IDO1 inhibitor therapy, chemotherapy, and radiation therapy in pediatric brain tumors. Twenty-nine patients enrolled in this trial showing a median PFS of 6.2 months, and the median time to regimen failure is 11.7 months (NCT02502708). There are also other ongoing clinical trials testing the IDO1 inhibitor combined with other therapies in malignant brain tumors (93) and rGBM (NCT03707457). Results from these trials are still pending. Furthermore, the efficacy and safety of these agents need to be evaluated in GBM patients.

Despite the great advances in treating hematological malignancies and solid tumors as well as promising results from preclinical and early-phase trials in GBM, immune checkpoint inhibitors have not yet demonstrated efficacy in GBM through large phase III clinical trials as a monotherapy or combination therapy with other treatments. The BBB should first be taken into account as it may block the antibody penetration into the CNS. Moreover, a tumor mutational burden that predicts the efficacy of immune checkpoint inhibitors across multiple solid tumors is actually associated with poor prognosis in glioma patients (94). Last but not least, the immunosuppressive TME and dynamic responses to tumorigenesis of GBM may also contribute to the obstacles faced by the immune checkpoint inhibitors. Thus, further investigations on the optimal combinations of multiple therapies as well as tumor genomic and immune characteristics are urgently required to clarify the role of checkpoint inhibitors in GBM in the future.

## Oncolytic Virotherapy

Oncolytic virotherapy (OV) employs naturally occurring or artificially engineered viruses, which are typically delivered intratumorally or postsurgically into the resection cavity to infect and lyse tumor cells, simultaneously triggering inflammation and immune responses to tumor cells and the virus (95). Multifarious virus species have been studied as oncolytic virus platforms for cancer therapy, such as herpes simplex virus (HSV), adenovirus, vaccinia virus, measles virus, poliovirus, and reovirus. In 2015, talimogene laherparepvec (T-VEC), a genetically modified HSV, was approved by the FDA for advanced melanoma as the first OV therapeutic in the United States (96). GBM virotherapy clinical trials started in 1991; Martuza et al. first reported engineered HSV for their capability of selective replication and killing of GBM cells (97). Since then, multifarious OVs have been tested in gliomas; however, they seldomly demonstrate efficacy in improving median OS in randomized trials (98). Here, we present evidence that OVs have recently been advanced to phase I/II trials in glioma patients, demonstrating remarkable efficacy in subsets of patients.

DNX-2401 (Ad5-Delta-24-RGD;tasadenoturev) is a replication-competent adenovirus with enhanced infectivity, high tumor selectivity, and a specific mutation to restrict viral replication. This virus can target integrins on GBM cells with a glycine/aspartic/arginine acid motif, which can increase infective specificity for tumor cells (99, 100). In a phase I trial of DNX-2401 (NCT00805376), 37 rGBM patients received a single intratumoral injection of DNX-2401 through the biopsy needle (cohort 1) or a permanently implanted catheter followed by tumor resection (cohort 2). In cohort 1, 20% of patients survived more than 3 years after treatment, and 3 patients showed more than 3 years of PFS with dramatic tumor reduction (95% or more, CR). Immunohistochemical analysis of post-treatment surgical specimens from cohort 2 revealed that DNX-2401 replicated and spread within the tumor and induced CD8^+^ and T-bet^+^ cell infiltration. No dose-limiting toxicities were observed, and adverse effects were reported in 15% of patients with no serious virus-related events of grade 3 or higher noted (101). Thus, this clinical trial, for the first time, showed direct oncolytic effects in GBM and provided evidence for elicitation of anti-GBM immune responses. In another phase I/II clinical trial that was initiated in 2010 for rGBM patients (NCT01582516), DNX-2401 was administered by catheters targeting the tumor mass and the surrounding infiltrated brain. Analysis of CSF from patients showed an elevated level of some cytokines that can increase the levels of CD64, a marker of M1-polarization, implying that DNX-2401 therapy can promote a macrophage phenotype shift from M2 to M1 (102). Currently, the combination of DNX-2401 treatment with pembrolizumab is under investigation in a phase II trial for rGBM patients (CAPTIVE/KEYNOTE-192, NCT02798406). Interim results were reported at the SNO 2018 annual meeting, including that the combinatorial therapy was well tolerated, and 100% 9-month survival for the first seven patients treated was noted (103). Publication of longer follow-up data is eagerly awaited.

The polio-rhinovirus chimera (PVS-RIPO) is a replication-competent, live attenuated poliovirus vaccine/human rhinovirus chimera that is engineered with a foreign (rhinovirus) ribosome entry site to ablate neurovirulence. PVS-RIPO can target the poliovirus receptor CD155 that is expressed on APC or overexpressed on tumor cells. In a phase I trial (NCT01491893), 61 patients with recurrent supratentorial grade IV malignant glioma received PVS-RIPO intratumorally by convection-enhanced delivery *via* a catheter. The patients who received PVS-RIPO had an OS rate of 21% at 24 and 36 months with two patients obtaining complete response and remaining alive for more than 70.4 months (104). A randomized phase II trial of PVS-RIPO solely or combined with lomustine in patients with recurrent grade IV malignant glioma (NCT02986178) is ongoing.

Other OVs, such as ParvOryx (oncolytic H-1 parvovirus), Toca 511 (a retroviral replication-competent vector), Reovirus, and HSV type 1 have also been tested in a phase I/II trial for GBM patients and obtained promising results (105–108). Although these early phase clinical trials demonstrate a survival benefit that OV has brought, these benefits were only appreciated by some subsets of patients with glioma. Recently, a comprehensive analysis of virotherapy trials for rGBM revealed that virotherapy can improve the 2- and 3-year survival rates compared with non-virotherapy clinical trials (2-year survival: 15% vs. 12%; 3-year survival rate: 9% vs. 6%) (109). Thus, further investigations and more large randomized controlled phase II/III trials need to be done to evaluate the benefit of OV.

## Conclusion

Current clinical trials of immunotherapy predominantly focus on the investigation of peptide vaccines, DC vaccines, CAR-T cells, checkpoint inhibitors, and OV. Many promising clinical outcomes have been achieved (110–116) however, immunotherapeutic successes in GBM are still lacking. Multiple factors challenge immunotherapy in GBM, including the immunosuppressive TME, tumor heterogeneity, tumor genomic characteristics, persistence and delivery of the vaccines, and efficiency of drug penetration through the BBB. Moreover, there remains a need for appropriate pre- and post-therapeutic biomarkers that may facilitate the establishment of a valid and standardized assessment for clinical efficacy in GBM. Immunotherapy for GBM requires integrated efforts with rational combinations of vaccine therapy, cell therapy, and radio- and chemotherapy, as well as molecule therapy targeting TME. These contributions promote the development of an optimal personalized therapeutic strategy for GBM patients.

## Author Contributions

All authors contributed to the study conception and design. Material preparation, data collection, and analysis were performed by BH, XL, YL, ZZ, and HZ. The first draft of the manuscript was written by BH and JZ, and all authors commented on previous versions of the manuscript. All authors contributed to the article and approved the submitted version.

## Funding

This program was funded by major science and technology special general projects of Jiangxi Province (20203BBGL73178).

## Conflict of Interest

The authors declare that the research was conducted in the absence of any commercial or financial relationships that could be construed as a potential conflict of interest.
